# Machine Learning Unveils Sphingolipid Metabolism's Role in Tumour Microenvironment and Immunotherapy in Lung Cancer

**DOI:** 10.1111/jcmm.70435

**Published:** 2025-03-30

**Authors:** Lili Xu, Jianchun Wu, Jianhui Tian, Bo Zhang, Yang Zhao, Zhenyu Zhao, Yingbin Luo, Yan Li

**Affiliations:** ^1^ Clinical Medical Center of Oncology, Shanghai Municipal Hospital of Traditional Chinese Medicine Shanghai University of Traditional Chinese Medicine Shanghai China; ^2^ Department of Emergency, Shanghai Municipal Hospital of Traditional Chinese Medicine Shanghai University of Traditional Chinese Medicine Shanghai China

**Keywords:** immune dynamics, immunotherapy, lung cancer, machine learning, sphingolipid metabolism, tumour microenvironment

## Abstract

TME is a core player in the development of a cancerous lesion, the immune evasive potential of the lesion, and its response to therapy. Sphingolipid metabolism, which governs a number of cellular processes, has been recognised as a player involved in the control of immune heterogeneity within the TME. Sphingolipid metabolism‐related genes prevalent in the TME of LUAD and LUSC were identified using transcriptomic analysis and clinical samples from the TCGA and GTEx databases. Lasso regression and survival SVM in the Etra Application were employed as machine learning algorithms to determine patient outcomes and to reveal key immune factors associated with gene expression and chemotherapeutic response. Gene expression in lung cancer cells was explored through scRNA‐seq data. Thereafter, mediation impact analysis was further performed to explain the defined relation between the immune cell subsets and sphingolipid metabolites and their risk impact on lung cancers. Genes involved in sphingolipid metabolism were dysregulated in lung cancer, correlating with immune cell infiltration and TME remodelling. Lasso regression identified ASAH1 and SMPD1 as strong prognostic markers. scRNA‐seq revealed higher gene expression in T cells, macrophages and fibroblasts. Sphingomyelin partially mediated the link between T lymphocyte abundance and lung cancer risk. High‐risk phenotypes exhibited enhanced immune evasion via altered regulatory T cell and macrophage polarisation. This research highlights the contribution of sphingolipid metabolism in shaping the TME and its implications for immunotherapy.

## 1 Introduction

Lung cancer, especially non‐small cell lung cancer (NSCLC), which includes lung adenocarcinoma (LUAD) and lung squamous cell carcinoma (LUSC), poses a significant global public health challenge due to its high incidence and mortality rates [1, 2, 3, 4]. The complexity of these tumours and their resistance to traditional treatments result in poor patient prognosis, prompting researchers to seek new therapeutic strategies and biomarkers. In recent years, the rise of immunotherapy has brought new hope to lung cancer treatment, with immune dynamics in the tumour microenvironment becoming a research hotspot [5, 6].

The sphingolipid metabolic pathway plays multiple roles in cell biology, including cell signalling, proliferation, apoptosis and differentiation. In oncology, the abnormal activation of sphingolipid metabolism is closely associated with tumour initiation, progression, invasion and metastasis. Particularly in immune regulation, sphingolipid metabolic products have been shown to influence the functions of immune cells, including T cells, B cells and natural killer (NK) cells, thereby promoting tumour immune evasion. For example, certain sphingolipids, such as sphingosine‐1‐phosphate (S1P), can regulate the migration and function of immune cells within the tumour microenvironment, impacting tumour immune surveillance. Additionally, key enzymes in sphingolipid metabolism, such as sphingosine kinases (SphKs) and ceramidases, play critical roles in tumour cell survival and proliferation. These findings highlight the significant role of sphingolipid metabolism in cancer biology, providing potential targets and biomarkers for the development of novel anti‐tumour therapeutic strategies [7, 8, 9].

This study employs machine learning techniques combined with large‐scale genomic and transcriptomic data and mediated mendelian randomization to deeply analyse the expression patterns of sphingolipid metabolism‐related genes in lung cancer and explore their impact on immune dynamics within the tumour microenvironment. By comparing the expression differences of sphingolipid metabolism genes in lung cancer tissues versus normal tissues, we aim to uncover their potential roles in lung cancer progression and assess their potential as prognostic biomarkers [10, 11, 12].

Machine learning has emerged as a powerful tool in cancer research, offering innovative approaches to analyse complex biological data. Techniques such as survival SVM, CoxBoost and Lasso regression allow for the integration and analysis of vast datasets, identifying patterns and relationships that may not be apparent through traditional methods [11, 12, 13]. In this study, machine learning models are applied to predict patient prognosis based on gene expression and clinical features and to explore the relationship between gene expression and drug sensitivity. These models can provide insights into potential therapeutic targets and personalised treatment strategies.

Furthermore, this study will explore the association between sphingolipid metabolism gene expression and clinical characteristics of lung cancer patients, including pathological stage, gender, age and specific gene expression levels, with the goal of discovering new predictive models for assessing patient prognosis and treatment response. Through these analyses, we hope to provide new perspectives for the precision treatment of lung cancer, particularly in the era of immunotherapy, offering patients more personalised treatment options. With the continuous advancement of immunotherapy and the integration of machine learning, these findings are expected to bring more effective and precise treatment options to lung cancer patients, improving their quality of life and survival outcomes.

## 2 Methods

### 2.1 Data Sources of Exposures and Outcomes

The GWAS summary statistics for each immunological feature can be obtained from the GWAS Catalogue (https://www.ebi.ac.uk/gwas/), with accession numbers ranging from GCST90001391 to GCST90002121. The analysis included a total of 731immunophenotypes. These immunophenotypes consisted of different types of measurements, including absolute cell counts, median fluorescence intensities that represent surface antigen levels, morphological parameters (MP) and relative cell counts. The AC (antigen presenting cells), MFI (mean fluorescence intensity) and RC (regulatory cells) features included B cells, CDCs (complement‐dependent cytotoxicity), mature stages of T cells, monocytes, myeloid cells, TBNK (T cells, B cells and natural killer cells) and Treg (regulatory T cells) panels. The MP feature included CDC (complete blood count) and TBNK (T‐cell, B‐cell and natural killer cell) panels. We obtained genome‐wide association summary statistics for metabolites by the FinnGen collaboration (https://www.finngen.fi/fi), with accession numbers for: GCST90199621‐GCST90201020. The summary association statistics of the exposure were derived from the GWAS data for lung cancer (ebi‐a‐GCST90018875) [24], which consisted of 3791 cases and 489,012 controls, and included information on 24, 188,684 SNPs. Both patient populations consisted exclusively of individuals of European descent [14, 15].

### 2.2 MR Analysis

Our major approach in Mendelian randomisation (MR) was the inverse‐variance weighted (IVW) method. Furthermore, we performed MR‐Egger, weighted median, weighted mode and simple mode analyses to verify the reliability and stability of our findings. Cochran's *Q* test was used to evaluate heterogeneity among summary estimates. In addition, we employed the MR‐Egger intercept to specifically handle and consider pleiotropy. In order to thoroughly evaluate the strength of our results, we conducted a leave‐one‐out analysis. The MR‐PRESSO test was utilised to detect and resolve pleiotropy, as well as exclude any data points that deviate significantly from the norm. Furthermore, we employed a two‐step MR analysis to identify mediator variables of metabolites that mediate the causal connection between immune cells and lung cancer. The two‐step MR analysis employed the equation β−β1 × β2 to quantify the direct effect of exposure on the outcome. Here, β represents the causal effect of the exposure on the outcome, β1 represents the causal effect of the exposure on the mediator, β2 represents the causal effect of the mediator on the outcome and β1 × β2 represents the mediating effect from the exposure to the outcome. The statistical analyses were performed using the ‘TwoSampleMR’ (version 0.5.7) packages and MR‐PRESSO package in the R statistical software (version 4.3.2). The *p*‐values less than 0.05 were selected to indicate statistical significance [16, 17, 18].

### 2.3 Study Population

A retrospective cohort study was conducted from January 2019 to December 2023, involving a total of 205 patients recruited from Shanghai Municipal Hospital of Traditional Chinese Medicine. The study included 137 patients who underwent surgical resection for early‐stage lung cancer in the past 6 months and were considered the observation group. The control group consisted of 58 patients who had pulmonary nodules with a diameter of ≤ 5 mm and were not yet eligible for surgery. We specifically eliminated individuals with other malignancies and autoimmune disorders from our study. In addition, 10 physically healthy individuals were also enlisted. The levels of their peripheral metabolites were established as a basis for comparison. Table 1 provided a comprehensive overview of the key attributes of each category of registered patients. The study protocol received approval from the Regional Ethics Committee at the Shanghai Municipal Hospital of Traditional Chinese Medicine. Every patient provided their informed consent, and this study was conducted in accordance with the Helsinki Declaration.

**TABLE 1:** The baseline clinical data of enrolled participants.

Characteristic	Observation group (*n* = 137)	Control group (*n* = 58)	Healthy group (*n* = 10)	*χ* ^2^	*p*
Gender					
Male	49	19	4	0.272	0.873
Female	88	39	6		
Age					
≤ 50	34	27	2	9.611	0.008
> 50	103	31	8		
Smoking history					
Yes	21	7	3	2.150	0.341
No	116	51	7		
Family history					
Yes	34	15	3	0.143	0.931
No	103	43	7		
Nodule diameter					
*n* ≤ 6 mm	0	50	/	/	/
6 mm < *n* ≤ 0.8 cm	76	8			
0.8 cm < *n* ≤ 2 cm	43	0			
2 cm < *n* ≤ 3 cm	18	0			

### 2.4 Sample Collection

The patients who were registered underwent fasting in the morning. A volume of 2 mL of venous blood was collected into a purple coagulation tube and then delivered to the laboratory department for the purpose of detecting peripheral blood T cell subsets. In addition, 2 mL of venous blood was drawn into the red procoagulant tube, gently shaken to make full contact with the tube wall, and left at 4°C for 30 min. Extract serum into a 15 mL sterile centrifuge tube and centrifuge at 1000 g at 4°C for 10 min; transfer the upper pale yellow serum to a 1.5 mL sterile centrifuge tube, label the sampling object (name, sample number, type and sampling time) and transfer to a −80°C freezer for storage. After collecting an adequate number of samples, they were sent to OE Biotechnology Co. Ltd., a reputable testing institution in Shanghai, China, for analysis.

### 2.5 Metabonomics Assays

Retrieve samples held at −80°C, allow them to thaw at ambient temperature, extract 100 μL of serum and introduce the internal standard (L‐2‐chlorophenylalanine, 0.3 mg/mL; Lyso PC17: 0, 0.01 mg/mL, all methanol formulations) in quantities of 10 μL each. Agitate the mixture vigorously for 10 s using a vortex. Dispense 300 μL of the protein precipitant solution, which consists of a mixture of methanol and acetonitrile in a volumetric ratio of 2:1. Proceed to mix the solution vigorously using a vortex mixer for a duration of 1 min. Perform ultrasonic extraction by immersing the sample in an ice water bath for a duration of 10 min. Allow to remain at a temperature of −20°C for a duration of 30 min. Centrifuge the mixture at 13000 rpm for 10 min at a temperature of 4°C. Take 300 μL of the liquid that settles at the bottom and remove the remaining liquid by evaporation. Next, mix the remaining liquid with 400 μL of a solution containing a mixture of methanol and water in a ratio of 1:4 (volume to volume). Agitate the mixture vigorously using a vortex mixer for 30 s, and then subject it to ultrasonic vibrations for 2 min. Spin the mixture in a centrifuge at 13,000 rpm for 10 min at a temperature of 4°C. Use a syringe to remove 150 μL of the liquid that settles at the top. Filter the liquid using a pinhole filter with a pore size of 0.22 μm. Transfer the filtered liquid to a vial for injection into a liquid chromatography‐mass spectrometry (LC–MS) system. Store the vial at a temperature of −80°C until it is ready for analysis. Quality control samples (QCs) are created by combining the extracts of all samples in equal amounts, ensuring that each QC has the same volume as the individual samples.

### 2.6 Single‐Cell Analysis

GSE131907: Single‐cell RNA sequencing (scRNA‐seq) for 208,506 cells derived from 58 lung adenocarcinomas from 44 patients, which covers primary tumour, lymph node and brain metastases and pleural effusion in addition to normal lung tissues and lymph nodes. During the data preprocessing stage, we implemented a series of detailed steps to ensure the quality and accuracy of RNA data. First, we validated RNA counts to ensure they met industry standards, including checking gene expression counts for each cell to ensure they were within a reasonable range. Next, we performed strict quality control on the cell data to remove low‐quality cells. Specifically, we calculated the proportion of mitochondrial gene expression in each cell and set a threshold (usually less than 10%) to remove cells with high mitochondrial gene expression, which could indicate cell damage or death. Additionally, we set thresholds for the number of genes detected and UMI counts, such as requiring each cell to have more than 250 detected genes and more than 500 UMI counts, to exclude cells with sparse gene expression or unstable data. Forms part‐preprocessing and PCA (principal component analysis) to characterise self‐similarity patterns within cell types or states. We then made PCA scatter plots showing a distribution of tumour samples corresponding to top principal components. This is followed by the use of t‐SNE (t‐distributed Stochastic Neighbor Embedding) to cluster cells, separate cell populations and endpoint plots for clustering to show variety in cell type or some potential subtypes. Cell–cell interaction network graphs are generated to explore the frequency of interactions between myeloid cells, TILs (T cells—red; NK and NKT populations combined), endothelial fibroblasts, stromal proliferating dendritic B IL‐3R dendritic cells, type 1 census population highlighting key cellular pathways involved in signal transduction [19, 20, 21].

### 2.7 GO/KEGG Analysis

When utilising R packages like limma or DESeq2 to identify differentially expressed genes (DEGs) from RNA‐Seq data, ensure that the results include gene identifiers (such as Entrez IDs or Gene Symbols) along with their respective statistical measures. The clusterProfiler R package is designed to facilitate this process, focusing on functional enrichment analysis. It covers Gene Ontology (GO) categories, which include Biological Process (BP), Molecular Function (MF) and Cellular Component (CC). For the enrichment analysis of KEGG pathways, the function enrichKEGG is employed [22].

### 2.8 Machine Learning Algorithm

Before model training, we conducted strict preprocessing on the data, including missing value handling, outlier detection and standardisation, to ensure the quality and consistency of the data. At the same time, we used the createDataPartition function to partition the data into training and testing sets, ensuring that the model is evaluated on independent testing sets and avoiding overfitting. The training set is used for model training and parameter tuning, while the test set is used for final model performance evaluation. This partitioning method can effectively test the generalisation ability of the model. In this example, we used the create DataPartition function from the caret package to divide the data into two datasets, training (50% of observations) and test. The train function from the caret package was used to train several machine learning models. We applied a function explain from the DALEX package for each model interpretation and predict to evaluate the models on the test set in terms of accuracy measured using ROC curves. The variable_importance function from the DALEX package was used to compute the importance of variables in each model. Lasso regression was also applied with the glmnet package [23, 24].

### 2.9 Immunomodulatory Molecules

Immunomodulatory agents play a pivotal role in cancer immunotherapy, with numerous molecules being explored for their potential to stimulate or suppress immune responses within the tumour microenvironment. Our study focused on examining the transcriptional profiles of these immunomodulatory factors and the epigenetic mechanisms that regulate their expression.

### 2.10 Analysis of Immune Cells

To maintain the accuracy and uniformity of the dataset, we gathered immune infiltration information for all TCGA samples using the publicly accessible TIMER 2.0 database. The heatmaps were then employed to graphically represent the Spearman correlation coefficients derived from our analysis, effectively illustrating the interrelations among various immune cell types [25].

### 2.11 Exploration of Targeted Drugs

In our quest to identify therapeutic strategies against gene‐driven tumorigenesis, we performed a connectivity map analysis (cMAP). This analysis led to the creation of a gene signature that highlighted the 150 genes with the greatest increase and the 150 genes with the greatest decrease in expression when comparing tumours with high and low levels of gene activity.

### 2.12 Statistical Analysis

First, we evaluated whether the required variables followed a normal distribution and showed homogeneity of variance. If these conditions were satisfied, the paired *t*‐test was used to assess differences across groups. The statistical analyses were conducted using R4.3.2. The *p*‐values less than 0.05 were considered statistically significant.

## 3 Results

### 3.1 The Expression Levels of Various Genes Related to Sphingolipid Metabolism in Different Types of Lung Cancer

**FIGURE 1:**
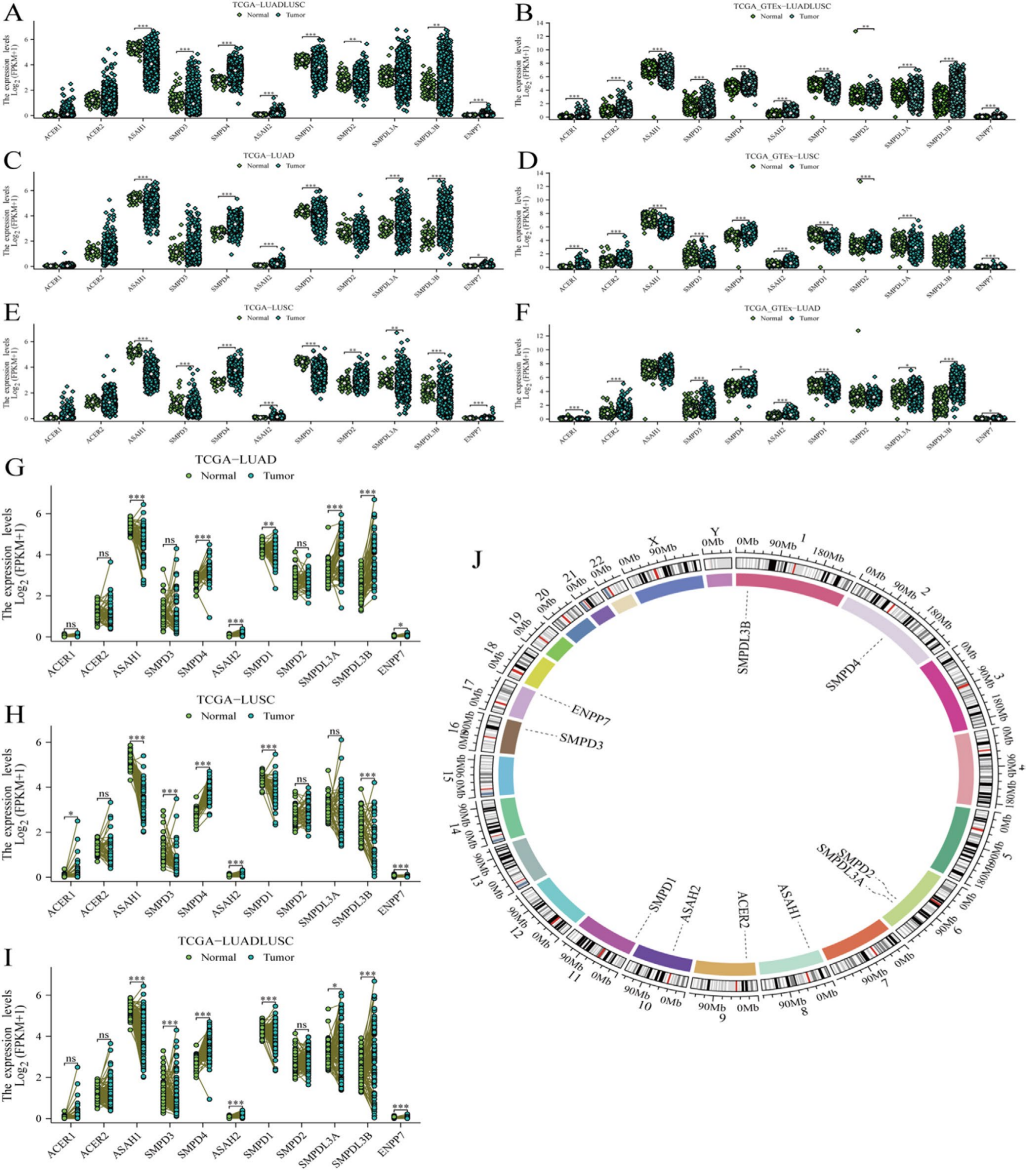
The expression levels of various genes related to sphingolipid metabolism in different types of lung cancer. (A) Compare gene expression differences between lung adenocarcinoma (LUAD) and normal tissue using TCGA data. (B) Analyse gene expression changes between LUAD and normal tissue using combined TCGA and GTEx data. (C) Compare gene expression differences between lung squamous cell carcinoma (LUSC) and normal tissue using TCGA data. (D) Compare gene expression between LUSC and normal tissue using combined TCGA and GTEx data. (E) Compare overall lung cancer (LC) gene expression with normal tissue using TCGA data. (F) Analyse overall lung cancer gene expression changes compared to normal tissue using combined TCGA and GTEx data. (G) Multiple genes in LUAD, such as ACER1, ASAH1 and SMPD1, are significantly upregulated or downregulated in tumour tissues. (H) Similar gene expression changes were also observed in LUSC, showing significant differences. (I) Combining data from LUAD and LUSC, consistent gene expression changes were demonstrated in lung cancer. (J) The circular chart displays the positions of these genes in the genome, providing visual information on their chromosome distribution.

Figure 1A compares gene expression in lung adenocarcinoma (LUAD) with normal tissue. Significant differences in expression are indicated by asterisks, with more asterisks representing higher statistical significance (e.g., *p < 0.05, **p < 0.01, ***p < 0.001). Certain genes show markedly higher or lower expression in tumour tissue compared to normal tissue. Figure 1B integrates data from TCGA and GTEx to compare LUAD with normal tissue. The pattern of gene expression differences is similar to panel A but with possibly more robust statistical validation due to combined datasets. Figure 1C focuses on lung squamous cell carcinoma (LUSC) compared to normal tissue. It highlights genes that are significantly dysregulated in LUSC, with some genes showing increased expression and others decreased. Figure 1D is similar to Figure 1C, but uses combined TCGA and GTEx data. The integration provides a comprehensive view of gene expression changes in LUSC. Figure 1E compares overall lung cancer (LC) gene expression with normal tissue. It aggregates data from different lung cancer subtypes, showing overall trends in gene expression changes. Figure 1F combines TCGA and GTEx data for a broader analysis of lung cancer versus normal tissue. It offers insights into the general dysregulation of sphingolipid metabolism genes in lung cancer. Figure 1G shows the expression levels of various genes in lung adenocarcinoma (LUAD) compared to normal tissue. Genes such as ACER1, ASAH1 and SMPD1 exhibit significant differences in expression between tumour and normal tissues. Figure 1H displays gene expression differences in lung squamous cell carcinoma (LUSC) versus normal tissue. Similar to LUAD, several genes show significant upregulation or downregulation in tumour samples. The expression patterns suggest distinct roles for these genes in LUSC pathogenesis. Figure 1I combines data from both LUAD and LUSC to provide an overall view of gene expression changes in lung cancer compared to normal tissue. The consistent expression changes across both cancer types highlight key genes potentially involved in lung cancer biology. Figure 1J provides a genomic view of the locations of the genes analysed in Figure 1G–I. Genes are mapped onto a circular representation of the human genome, indicating their chromosomal positions. This visualisation helps identify potential genomic regions of interest and their relation to gene expression changes.

### 3.2 The Analysis of Gene Expression Data Related to Lung Cancer Prognosis Using Machine Learning Methods

**FIGURE 2:**
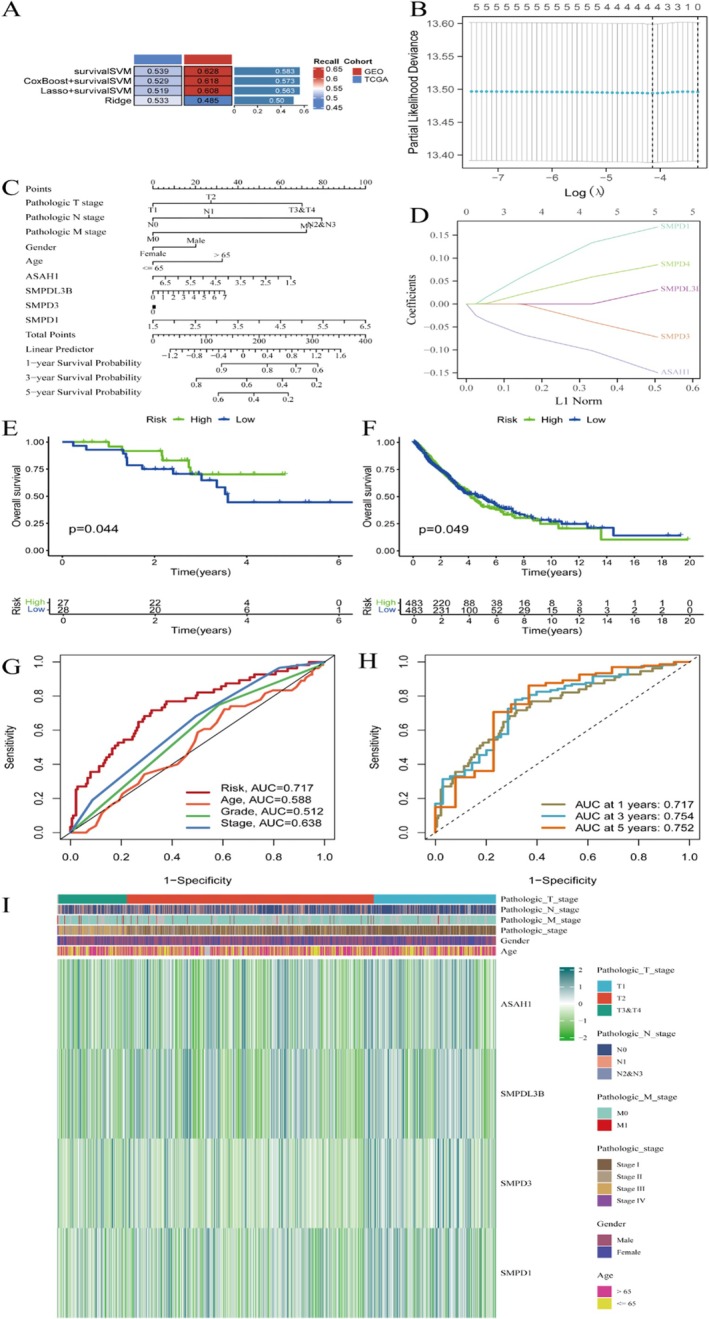
The analysis of gene expression data related to lung cancer prognosis using machine learning methods. (A) Heatmap showing survival model performance across different cohorts. (B) Plot for selecting the optimal lambda in a regression model. (C) Nomogram for predicting patient survival based on various clinical factors. (D) Coefficient paths for genes in a regression model. (E) Kaplan–Meier survival curve comparing high‐and low‐risk groups, with a significant *p*‐value. (F) Another Kaplan–Meier curve for a different dataset or subgroup, also showing significant results. (G) ROC curves assessing the predictive accuracy of risk scores and clinical features, with AUC values. (H) Time‐dependent ROC curves showing model performance at different years. (I) Heatmap displaying gene expression and clinical data across samples, highlighting different risk groups and clinical features.

Figure 2A presents a heatmap showing the performance of various survival models across different cohorts, highlighting the predictive accuracy with distinct colour intensities. Figure 2B illustrates the process of selecting the optimal lambda for a regression model, which is crucial for balancing model complexity and performance. Figure 2C features a nomogram that predicts patient survival based on clinical factors such as age, stage and gene expression, providing a visual tool for individualised risk assessment. Figure 2D shows the coefficient paths for genes in a regression model, indicating how gene importance changes with different levels of regularisation. Figure 2E,F depicts Kaplan–Meier survival curves, comparing high and low‐risk groups with significant p‐values, suggesting a meaningful difference in survival outcomes between these groups. Figure 2G presents ROC curves assessing the predictive accuracy of risk scores and clinical features, with AUC values indicating the model's ability to distinguish between different risk levels. Figure 2H shows time‐dependent ROC curves that evaluate model performance over 1, 3 and 5 years, demonstrating consistent predictive ability across time points. Finally, Figure 2I is a comprehensive heatmap displaying gene expression and clinical data across samples, highlighting variations in expression levels and clinical features such as pathological stage and gender, thereby providing a holistic view of the data and aiding in the identification of high‐risk patient groups.

### 3.3 Analysis of Lung Cancer Patients From the TCGA Cohort

**FIGURE 3:**
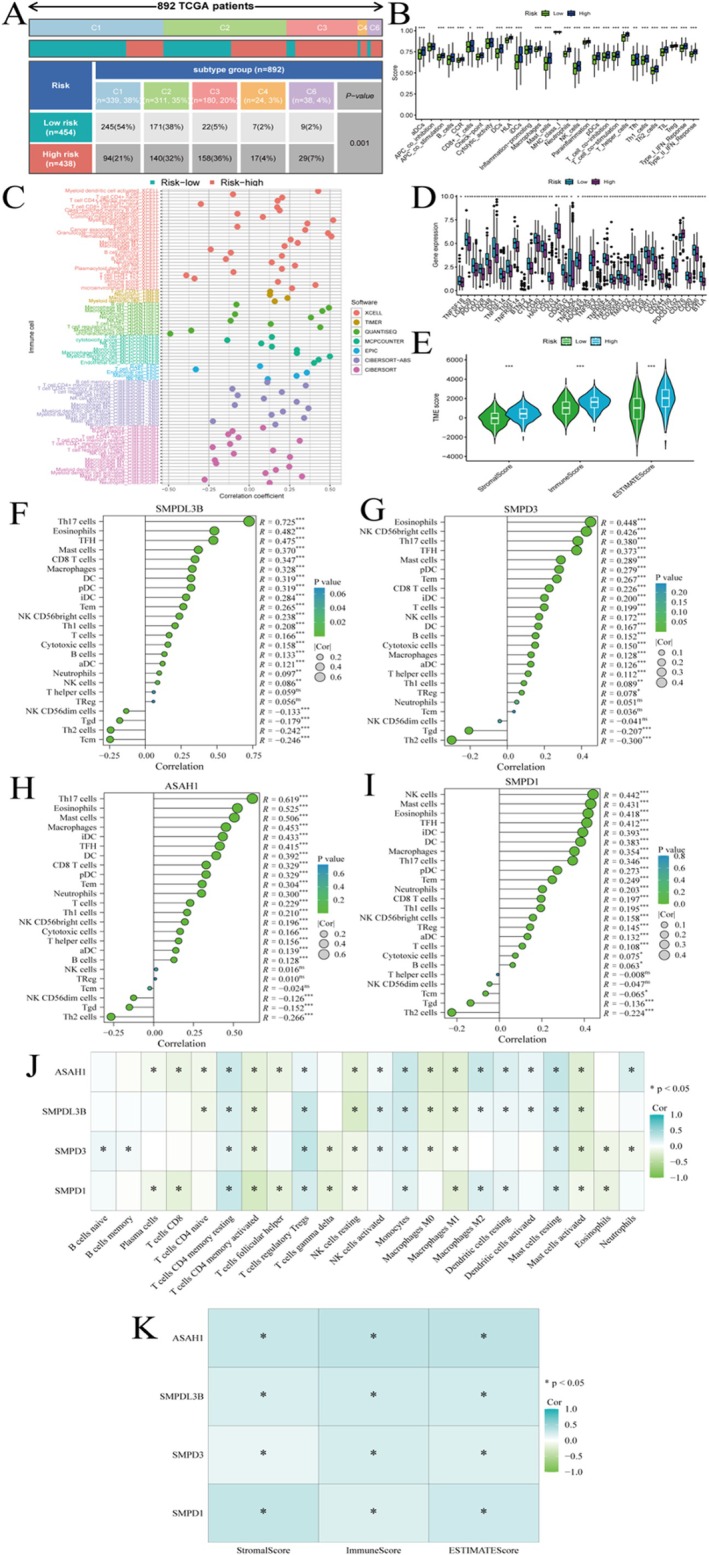
Analysis of lung cancer patients from the TCGA cohort. (A) Patients are classified by molecular subtypes (C1–C6) and risk groups (low‐risk and high‐risk), with a significant association between subtype and risk. (B) Correlation of different immune cell types in high‐risk and low‐risk groups, showing distribution differences between the two groups. (C) Expression differences of specific genes between high‐risk and low‐risk groups, revealing potential biomarkers. (D) Significant differences in immune cell infiltration levels between high‐risk and low‐risk groups. (E) Higher TIDE scores in high‐risk groups indicate stronger immune evasion and dysfunction. (F–I) Genes (SMPDL3B, SMPD3, ASAH1 and SMPD1) show significant positive and negative correlations with different immune cells (such as Th17 cells, eosinophils and NK cells). (J) The heatmap displays the correlation coefficients between each gene and various immune cell types, with colour intensity indicating correlation strength. (K) Correlations between gene expression and immune, stromal and ESTIMATE scores reflect the genes' impact on the tumour microenvironment.

Figure 3A shows that 892 TCGA patients are categorised into different subtypes (C1–C6) based on molecular characteristics. Patients are further divided into low‐risk (454 patients) and high‐risk (438 patients) groups. The distribution of subtypes across risk groups is shown, with a significant *p*‐value (0.001) indicating a strong association between subtype and risk. Figure 3B: Correlation of immune cells shows the correlation between various immune cell types and risk groups. Different colours represent different software tools used for immune cell estimation. The plot highlights which immune cells are more prevalent in low‐risk versus high‐risk groups. Figure 3C displays expression differences of specific genes between high‐risk and low‐risk groups. Significant differences are noted indicating genes that may be associated with risk stratification. Figure 3D illustrates the levels of immune cell infiltration in high‐risk versus low‐risk groups. Significant differences suggest varying immune environments between the two risk categories. Figure 3E represents TIDE scores for high‐risk and low‐risk groups. Higher TIDE scores in high‐risk groups suggest greater immune evasion and dysfunction. Figure 3F shows SMPDL3B gene‐positive correlations with Th17 cells, eosinophils and mast cells. Negative correlations with Th2 cells and Tregs. Significant correlations are marked by asterisks, with colour and size indicating p‐values and correlation strength. Figure 3G shows SMPD3 gene positively correlated with eosinophils and NK CD56bright cells. Negatively correlated with T cells and Th2 cells. Figure 3H shows ASAH1 positive correlations with Th17 cells, eosinophils and mast cells. Negative correlations with Th2 cells and Tregs. Figure 3I shows SMPD1 positive correlations with NK cells, mast cells and eosinophils. Negative correlations with Th2 cells and Tregs. Figure 3J displays the correlation coefficients between each gene and various immune cell types. Colour intensity reflects the strength and direction of the correlation. Figure 3K examines the correlation between gene expression and stromal, immune and ESTIMATE scores. These scores represent the tumour microenvironment's stromal and immune components.

### 3.4 The Relationship Between Copy Number Variations (CNVs), mRNA Expression and Immune Cell Abundance in Lung Cancer

**FIGURE 4:**
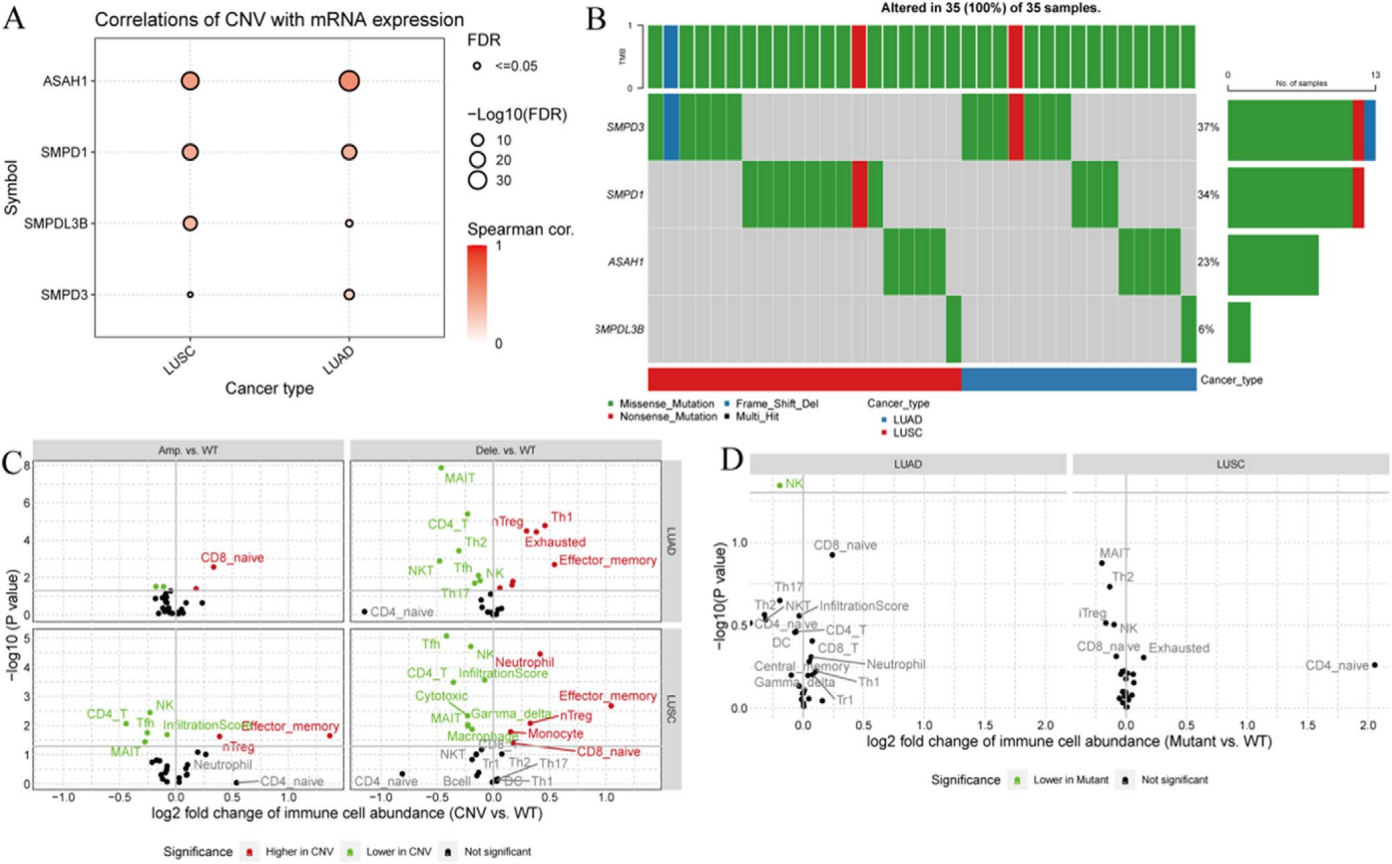
The relationship between copy number variations (CNVs), mRNA expression and immune cell abundance in lung cancer. (A) CNVs are significantly correlated with mRNA expression, indicating that changes in gene dosage may affect gene expression levels. (B) The CNV changes of each gene in 35 samples were displayed showing different types of gene changes (such as amplification and deletion). (C) The changes in immune cell abundance compared to wild‐type (WT) samples indicate the impact of CNV on immune cell infiltration. (D) The changes in immune cell abundance compared to WT samples indicate the impact of mutations on immune responses.

Figure 4A shows correlation of CNV with mRNA expression displays the correlation between CNVs and mRNA expression for genes (ASAH1, SMPD1, SMPDL3B and SMPD3) in LUAD and LUSC. The size of the circles represents the significance level (FDR), while the colour indicates the strength of the Spearman correlation. Significant correlations suggest that CNVs might influence gene expression levels.

Figure 4B shows CNV alterations in samples illustrate CNV alterations across 35 samples, showing the percentage of samples with alterations for each gene. The bar chart on the right shows the proportion of alterations for each gene, indicating which genes are most frequently altered. Figure 4C shows immune cell abundance changes show the log_2_ fold change of immune cell abundance between CNV‐altered and wild‐type (WT) samples for LUAD and LUSC. Red and green colours indicate significant increases or decreases in immune cell types, respectively. Highlights how CNV alterations in genes can impact immune cell infiltration.

**FIGURE 5:**
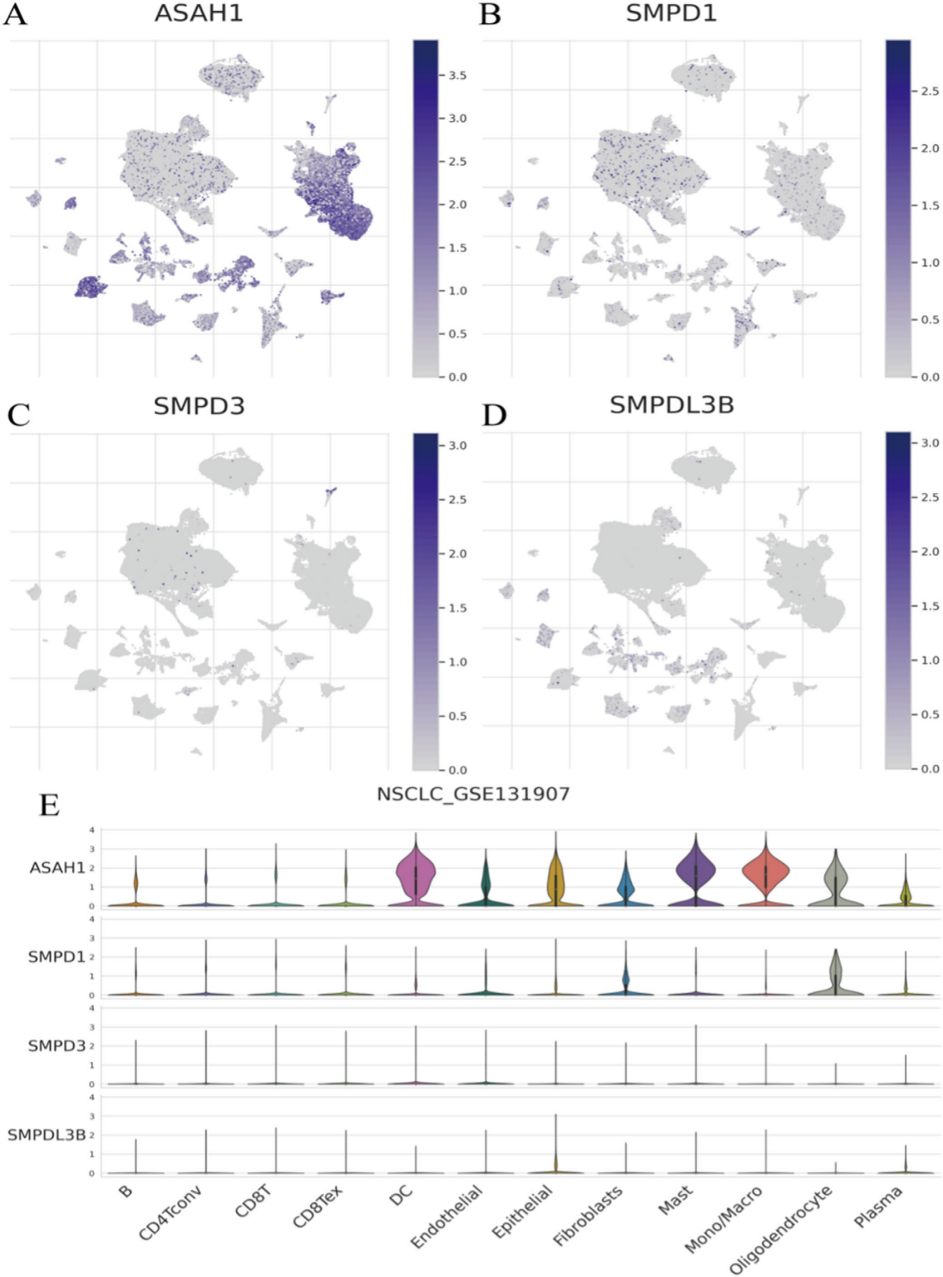
The expression patterns of four genes in the NSCLC_GSE131907 dataset. (A–D) Gene expression maps. ASAH1: High expression in specific clusters. SMPD1: Moderate expression across several clusters. SMPD3 and SMPDL3B: Low expression levels. (E) Violin Plots by Cell Type. ASAH1: High in fibroblasts, epithelial cells and monocytes/macrophages. SMPD1: Moderate in endothelial and epithelial cells. SMPD3 and SMPDL3B: Low across most cell types.

Figure 4D shows similar scatter plots as Figure 5C, but comparing mutant versus WT samples. Significant changes are highlighted, suggesting specific immune responses associated with genetic alterations.

### 3.5 The Correlation Between mRNA Expression of Specific Genes and Drug Sensitivity in Two Datasets: CTRP and GDSC

**FIGURE 6:**
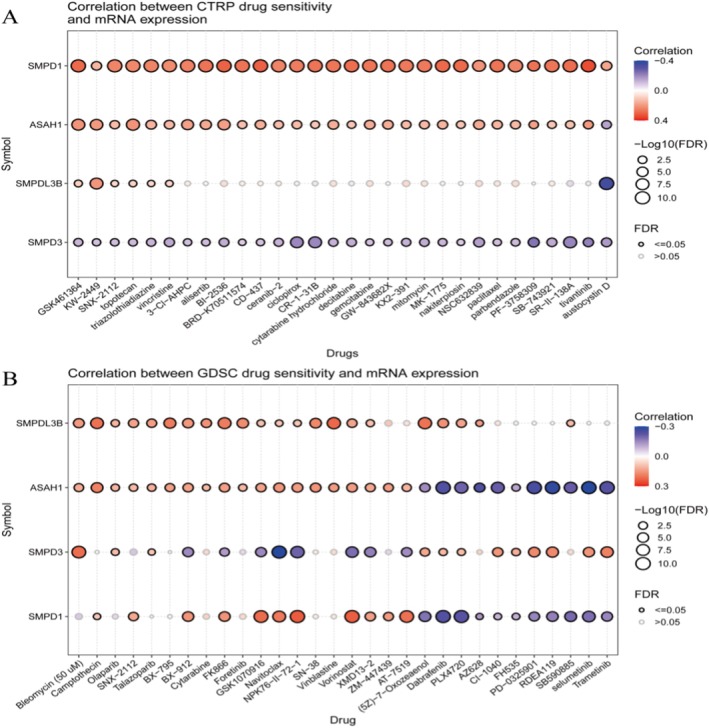
The correlation between mRNA expression of specific genes and drug sensitivity in two datasets: CTRP and GDSC. (A) In the CTRP dataset, genes such as SMPD1 and ASAH1 show significant correlations with sensitivity to various drugs, indicating their potential predictive value. (B) In the GDSC dataset, similar analysis shows significant correlations between SMPDL3B and ASAH1 with certain drugs.

Figure 6A shows CTRP drug sensitivity correlation shows the correlation between mRNA expression of genes (SMPD1, ASAH1, SMPDL3B and SMPD3) and sensitivity to various drugs. The size of the circles represents the significance level (FDR), while the colour indicates the correlation strength (red for positive, blue for negative). SMPD1 and ASAH1 show strong correlations with several drugs, indicating potential predictive value for drug response. Figure 6B shows similar analysis as Figure 6A but using the GDSC dataset. Again, the size and colour of the circles denote significance and correlation strength. Notable correlations are observed for SMPDL3B and ASAH1 with certain drugs, suggesting these genes might influence drug efficacy.

### 3.6 Analysis of Single‐Cell RNA Sequencing Data From the NSCLC_GSE131907 Dataset

**FIGURE 7:**
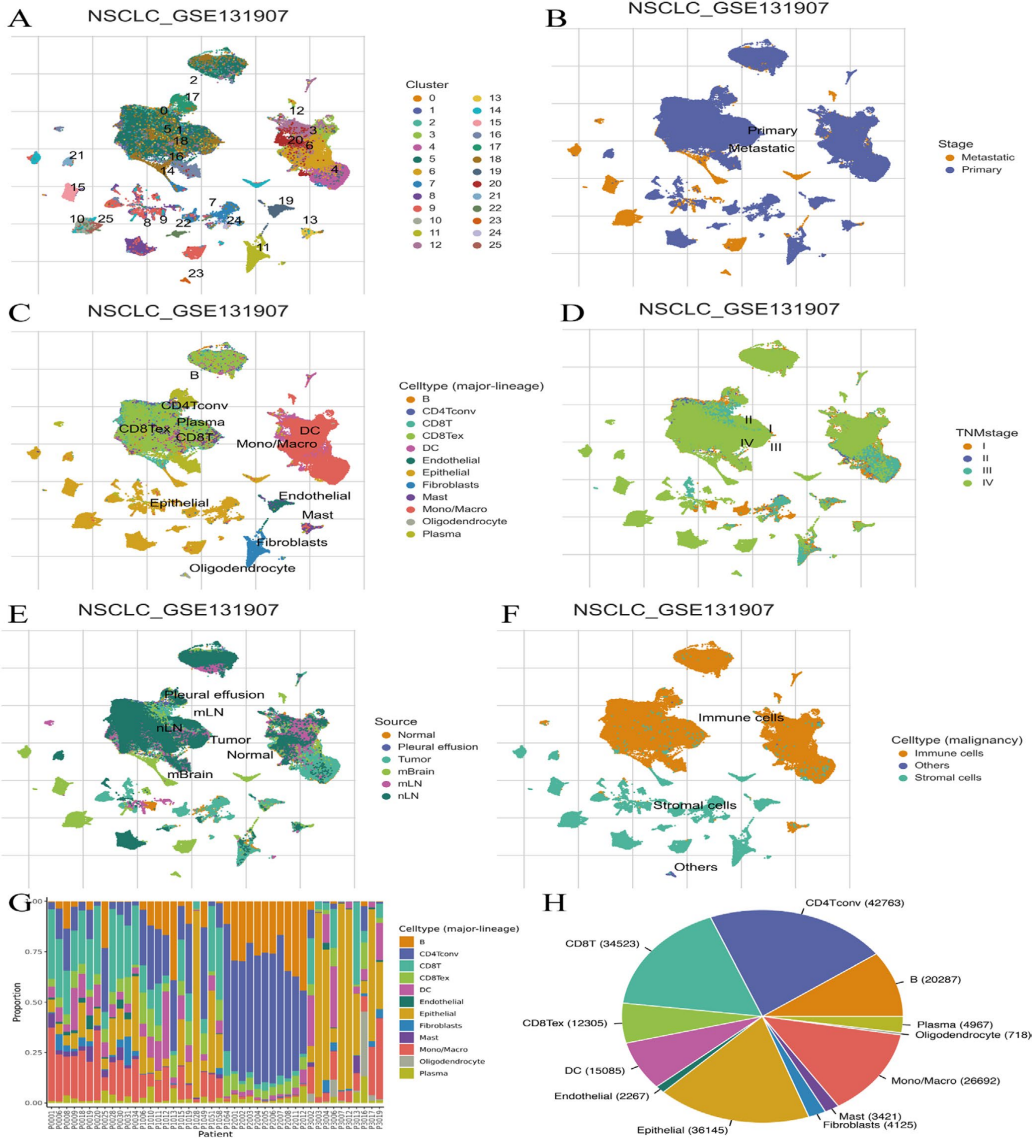
Analysis of single‐cell RNA sequencing data from the NSCLC_GSE131907 dataset. (A) Cells are grouped into clusters (0–25) based on gene expression, showing diverse cell populations. (B) Cells are labelled as primary or metastatic, illustrating their distribution across clusters. (C) Cells are annotated with specific types, such as CD8 T cells and fibroblasts, highlighting the tumour microenvironment's diversity. (D) Cells are categorised by TNM stage (I–IV), showing variation in cell populations with cancer progression. (E) Cells are marked by their origin, such as tumour or normal tissue, indicating the source distribution. (F) Cells are classified into immune, stromal and other categories, providing insights into the tumour microenvironment composition. (G) A stacked bar chart shows the proportion of various cell types for each patient. There is significant variability, indicating heterogeneity in the tumour microenvironment. (H) A pie chart displays the overall distribution of major cell types across all patients. Dominant cell types include CD4 T cells, epithelial cells and monocytes/macrophages.

Figure 7A shows that cells are grouped into distinct clusters (0–25) based on gene expression profiles. Each cluster is colour‐coded, representing different cell populations or states within the dataset. Figure 7B shows that cells are labelled according to their stage: primary or metastatic. Primary cells are shown in blue, while metastatic cells are in orange, illustrating the distribution of stages across clusters.

Figure 7C shows that cells are annotated with specific cell types (e.g., CD8 T cells, fibroblasts and endothelial cells). The plot highlights the diversity of cell types present in the tumour microenvironment. Figure 7D shows that cells are categorised by TNM stage (I–IV). Different colours represent each stage, showing how cell populations vary with cancer progression. Figure 7E shows that cells are marked by their source, such as tumour, normal tissue, pleural effusion and lymph nodes (mLN and nLN). This highlights the origin of the cells and their distribution in the dataset. Figure 7F shows that cells are classified into immune cells, stromal cells and others. The distribution of these categories provides insights into the tumour microenvironment's composition. Figure 7G shows a stacked bar chart illustrating the proportion of different cell types for each patient. Each colour represents a specific cell type, such as CD4 T cells, CD8 T cells, B cells, dendritic cells (DC), epithelial cells and more. The chart highlights the variability in cell type composition among different patients, indicating heterogeneity in the tumour microenvironment. Figure 7H shows a pie chart shows the overall distribution of major cell types across all patients. The most abundant cell types include CD4 T cells, epithelial cells, CD8 T cells and monocytes/macrophages. Each segment is labelled with the cell type and the total number of cells identified, providing a clear overview of cell prevalence.

### 3.7 The Expression Patterns of Four Genes in the NSCLC_GSE131907 Dataset

ASAH1 (Figure 5A) shows high expression in specific clusters, indicating its presence in particular cell populations. SMPD1 (Figure 5B) exhibits moderate expression across several clusters, suggesting a broader distribution. SMPD3 (Figure 5C) displays low expression, with limited presence in the dataset. SMPDL3B (Figure 5D), similar to SMPD3, shows low expression levels. ASAH1: high expression in fibroblasts, epithelial cells and monocytes/macrophages, indicating its role in these cell types. SMPD1: moderate expression in endothelial and epithelial cells, suggesting involvement in these tissues. SMPD3 and SMPDL3B: low expression across most cell types, indicating limited activity (Figure 5E).

### 3.8 Analyses of Gene Expression Across Different Conditions in the NSCLC_GSE131907 Dataset

**FIGURE 8:**
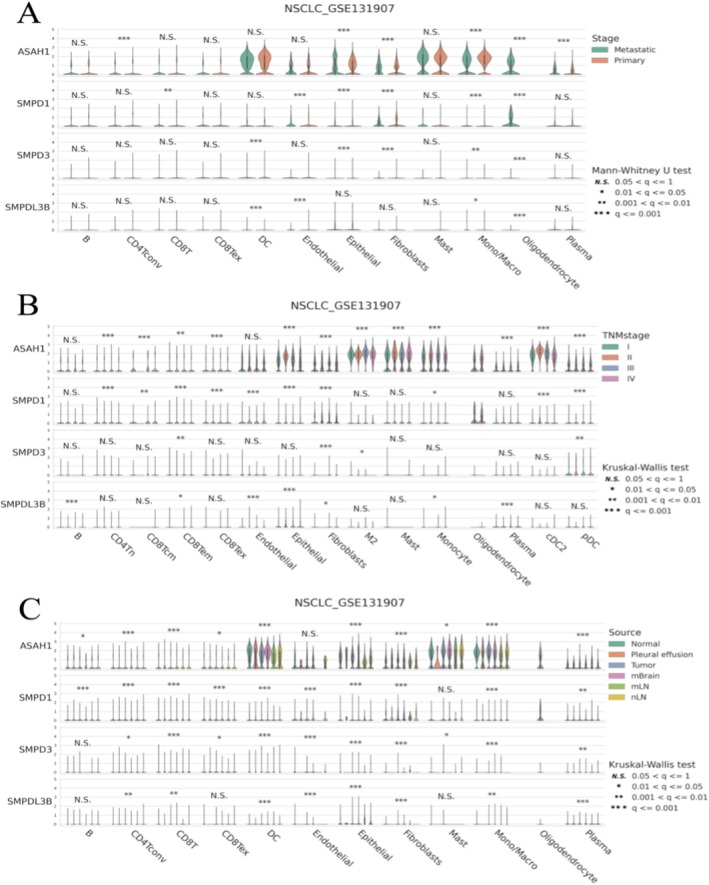
Analyses of gene expression across different conditions in the NSCLC_GSE131907 dataset. (A) Expression by Cancer Stage. (B) Expression by TNM Stage. (C) Expression by Sample Source. ASAH1 and SMPD1 show significant variations across stages and sources, suggesting important roles in tumour biology. SMPD3 and SMPDL3B have lower and less variable expression.

Figure 8A shows ASAH1: higher expression in primary tumours, especially in fibroblasts and epithelial cells. SMPD1: Significant differences in expression between stages in several cell types. SMPD3 and SMPDL3B: Generally low expression with fewer significant differences. Figure 8B shows ASAH1: expression varies significantly with TNM stage, particularly in fibroblasts and epithelial cells. SMPD1: Shows stage‐dependent expression patterns in certain cell types. SMPD3 and SMPDL3B: Limited significant variations across stages. Figure 8C shows ASAH1: highest expression in tumour samples, especially in fibroblasts and epithelial cells. SMPD1: significant differences in expression across sources for multiple cell types. SMPD3 and SMPDL3B: minimal expression differences based on sample source.

### 3.9 Mediating Effect

**FIGURE 9:**
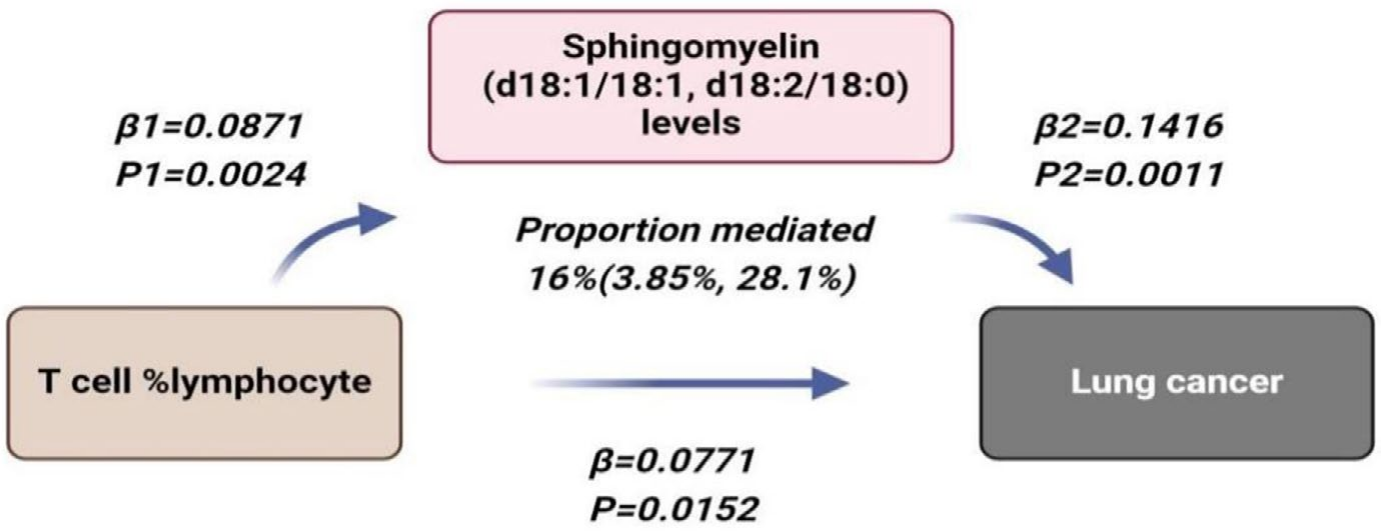
Mediation effect of Sphingomyelin levels in the causal association between T cell %lymphocyte and lung cancer. The figure shows that the risk of lung cancer is increased when the abundance of T cell %lymphocyte increases, with an effect size of 0.0771. Sphingomyelin levels increased with increasing abundance of T cell %lymphocyte, with an effect value of 0.0871. The risk of lung cancer increased with an increase in Sphingomyelin levels, with an effect size of 0.1416. The association between T cell %lymphocyte and risk of lung cancer is mediated by Sphingomyelin levels. (proportion mediated = 16%)].

**FIGURE 10:**
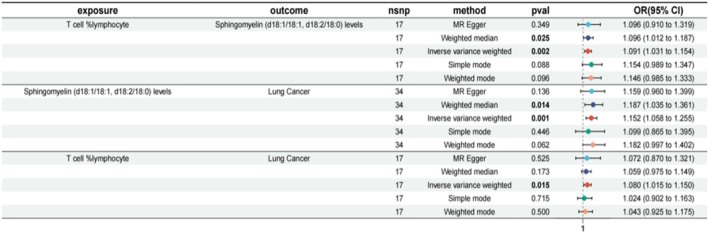
Forest plots of the mediating role of Sphingomyelin levels in the causal associations of T cell %lymphocyte and lung cancer. The figure summarises the analysis of the association between T cell sphingolipids and outcomes like sphingomyelin levels and lung cancer. Various methods, including MR Egger and weighted median, are used to calculate odds ratios (OR) and confidence intervals (CI). Significant *p*‐values suggest potential associations, with OR values indicating the strength and direction of these associations.

Therefore, we investigated the role of Sphingomyelin as the mediator connecting T cell %lymphocyte and the risk of lung cancer (Figure 9). Employing a two‐step MR analysis, we determined that there is a potential mediating impact of Sphingomyelin levels. The mediated effect was 0.0123, with a mediated proportion of 16% (Figure 10).

### 3.10 Association Between T Cell %Lymphocyte and Lung Cancer

**FIGURE 11:**
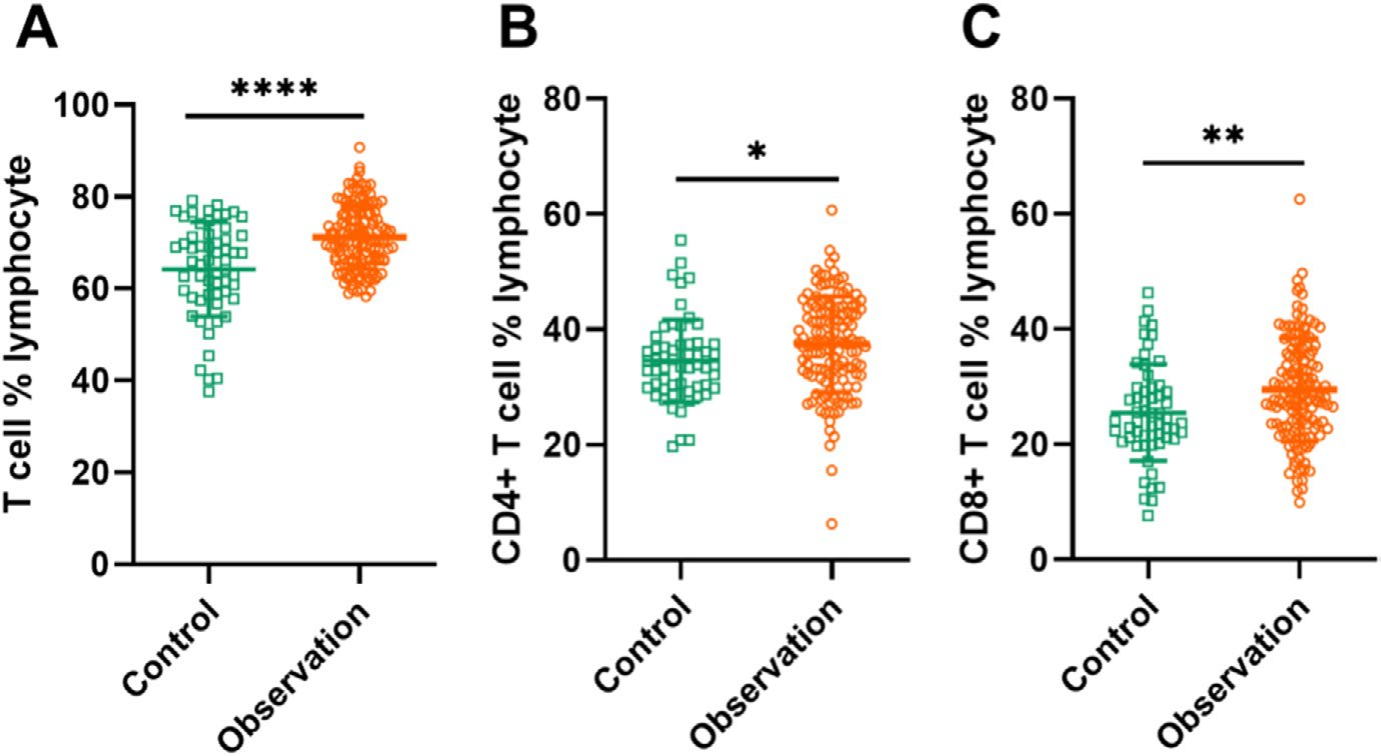
Percentage of CD3+, CD4+ and CD8+ in Peripheral blood between the control group and the observation group. (A) Shows a scatter plot comparing two groups with distinct distributions. (B) Another scatter plot illustrating differences in data spread between the groups. (C) A final scatter plot highlighting variability and potential differences in the data points between the two groups. * p¼0.05, ** *p* < 0.01, *** *p* < 0.001, **** *p* < 0.0001 in either corresponding text or figure.

Initially, we compared the fundamental characteristics of the patients in both groups, as well as the healthy controls. Flow cytometry was performed on the peripheral blood T lymphocytes of the enrolled patients. The findings (Figure 11A) of our study demonstrated a statistically significant increase in the percentage of T lymphocytes in the peripheral blood of the observation group compared to the control group (71.18% ± 6.89% vs. 64.24% ± 10.27%). In terms of specifics, the observation group exhibited a notably larger proportion of helper T cells (CD4 + T cell) in their peripheral blood compared to the control group (37.43% ± 8.27% vs. 34.55% ± 7.14%) (Figure 11B). In addition, the observation group had a considerably larger ratio of suppressor T cells (CD8+ T cells) in their peripheral blood compared to the control group (29.49% ± 8.98% vs. 25.51% ± 8.37%) (Figure 11C).

## 4 Discussion

In this study, we utilised machine learning techniques combined with big data analysis to explore the role of sphingolipid metabolism in lung cancer, particularly its impact on immune infiltration. The following is a detailed discussion of the findings, highlighting the roles of machine learning and immune infiltration in lung cancer.

Machine learning, as an advanced data analysis tool, is increasingly applied in biomedical research. In lung cancer studies, machine learning models have been used to identify biomarkers, predict prognosis, classify tumour subtypes and develop personalised treatment plans. In this study, we employed various machine learning models, including survival SVM, CoxBoost and Lasso regression, to analyse survival data and gene expression data of lung cancer patients [23, 24, 26, 27]. One of the main advantages of survival SVM is its ability to effectively handle right‐censored data, which is crucial for survival analysis. Additionally, it performs well in dealing with high‐dimensional data, making it suitable for situations where the number of features is much larger than the number of samples. Survival SVM also offers a variety of kernel function options, such as linear and radial basis function (RBF) kernels, allowing it to adapt to different data characteristics and requirements. However, the model also has some drawbacks. For instance, it has a high computational complexity, especially when dealing with large‐scale datasets, which can result in longer training times. Moreover, the model's performance is highly sensitive to the choice of kernel functions and parameter settings, requiring meticulous tuning, such as selecting appropriate values for the penalty coefficient *C* and the RBF kernel's γ.

These models not only enhanced the accuracy of lung cancer prognosis predictions but also helped identify key genes closely related to prognosis. Specifically, the Lasso regression model reduced model complexity through regularisation, improving prediction stability and accuracy. This finding demonstrates that machine learning models can effectively handle complex biomedical data, providing new strategies for precision medicine in lung cancer. Additionally, the predictive power of machine learning models can be further enhanced by integrating multi‐source data, as demonstrated in this study through the integration of TCGA and GTEx database data.

Immune infiltration is a crucial component of the tumour microenvironment, influencing tumour growth, metastasis and response to treatment. In lung cancer, the composition and state of immune cells are closely related to tumour progression and prognosis [28, 29, 30]. Our results indicate that the expression of sphingolipid metabolism‐related genes is closely linked to the level of immune cell infiltration in lung cancer. Specifically, we found that certain sphingolipid metabolism genes are positively correlated with specific immune cell subsets, such as Th17 cells, eosinophils and mast cells, while negatively correlated with Th2 cells and regulatory T cells (Tregs). These results reveal the potential role of sphingolipid metabolism in regulating the tumour immune microenvironment, possibly by influencing the function and distribution of immune cells to promote or inhibit tumour development.

Our study also explored the role of sphingolipid metabolism in immune evasion in lung cancer. By analysing differences in immune cell infiltration between high‐risk and low‐risk lung cancer patients, we found that immune evasion and dysfunction were more pronounced in the high‐risk group. This may be due to sphingolipid metabolism products regulating immune cell activity, thus affecting the tumour's resistance to immune attack. These findings provide a theoretical basis for developing immunotherapy strategies targeting sphingolipid metabolism. Additionally, our study found that the expression of sphingolipid metabolism genes is related to tumour TNM stage and sample origin, further emphasising their important role in lung cancer progression.

Key enzymes in the sphingolipid metabolic pathway, such as sphingosine kinases (SphKs) and ceramidases, play critical roles in tumour cell survival and proliferation. Specific small‐molecule inhibitors or antibodies targeting the activity of these enzymes can be developed to block the sphingolipid metabolic pathway, thereby inhibiting tumour cell growth and spread. For example, SphK1 inhibitors have been shown to suppress tumour cell proliferation and migration while enhancing the efficacy of chemotherapy drugs. There is a close regulatory relationship between sphingolipid metabolism and lipid metabolism. For example, certain enzymes in the sphingolipid metabolic pathway, such as ceramidase, can convert ceramide into sphingosine, which can then be further converted into sphingosine‐1‐phosphate (S1P). These metabolites play important roles in lipid metabolism. Studying the interactions between these metabolic pathways can provide a more comprehensive understanding of the dynamic balance of lipids within cells and their roles in diseases.

Our findings have potential implications for lung cancer treatment strategies. By identifying sphingolipid metabolism genes closely related to prognosis, we can offer more personalised treatment plans for patients. Furthermore, by analysing the relationship between gene expression and drug sensitivity, we may be able to predict patient responses to specific drugs, achieving precision medicine. These findings highlight the importance of considering the immune microenvironment and metabolic pathways in lung cancer treatment. Future treatment strategies may include targeted therapies against these key metabolic pathways, as well as methods to enhance immunotherapy effects by modulating the immune microenvironment.

## 5 Limitations

The performance of machine learning models heavily depends on the quality and quantity of training data. Although we integrated data from the TCGA and GTEx databases, these datasets may have limitations, such as insufficient sample sizes and a lack of diversity. If the training data do not comprehensively cover the various types and clinical features of lung cancer, the model's generalisation ability in real‐world applications might be limited, leading to decreased prediction accuracy for new data. Additionally, some machine learning models, like survival SVM and CoxBoost, exhibit a certain black‐box nature, making their internal decision processes difficult to interpret intuitively. This makes it challenging to understand how the model predicts lung cancer prognosis based on sphingolipid metabolism gene expression data and immune cell infiltration characteristics. It limits the model's credibility and transparency and affects clinicians' ability to understand and apply the model's predictions.

## 6 Conclusion

In summary, this study utilised machine learning techniques to uncover the role of sphingolipid metabolism in lung cancer, particularly its impact on immune infiltration. These findings offer the potential to develop novel biomarkers and therapeutic targets based on sphingolipid metabolism, with the aim of improving the accuracy of lung cancer diagnosis and the effectiveness of treatment.

## Author Contributions

**Lili Xu:** conceptualization (equal), data curation (equal), writing – original draft (equal). **Jianchun Wu:** conceptualization (equal), data curation (equal), writing – original draft (equal). **Jianhui Tian:** formal analysis (equal), validation (equal). **Bo Zhang:** investigation (equal), software (equal). **Yang Zhao:** investigation (equal), software (equal). **Zhenyu Zhao:** investigation (equal), supervision (equal). **Yingbin Luo:** project administration (equal), writing – review and editing (equal). **Yan Li:** funding acquisition (equal), project administration (equal), writing – review and editing (equal).

## Conflicts of Interest

The authors declare no conflicts of interest.

## Data Availability

The data used in this study are publicly available in the TCGA, GTEx, and GEO (GSE131907) databases. GWAS summary statistics are accessible via the GWAS Catalog and the FinnGen consortium. For further inquiries, please contact the corresponding authors.
